# Efficient Conditions of Enzyme-Assisted Extractions and Pressurized Liquids for Recovering Polyphenols with Antioxidant Capacity from Pisco Grape Pomace as a Sustainable Strategy

**DOI:** 10.3390/molecules30142977

**Published:** 2025-07-15

**Authors:** Jacqueline Poblete, Mario Aranda, Issis Quispe-Fuentes

**Affiliations:** 1 Food Engineering Department, Universidad de La Serena, Av. Raúl Bitrán 1305, La Serena 1700000, Chile; j.pobletegalleguillos@gmail.com; 2 Department of Chemistry and Pharmacy, Pontificia Universidad Católica de Chile, Santiago 7810000, Chile; mario.aranda@uc.cl

**Keywords:** pisco grape pomace, efficient conditions, enzyme-assisted extraction, liquid pressurized extraction, polyphenols, antioxidant capacity

## Abstract

The pisco industry generates significant environmental waste, particularly grape pomace, which is a rich source of phenolic compounds. Emerging extraction technologies offer promising alternatives for recovering these bioactive components. This study evaluated enzyme-assisted extraction (EAE) and pressurized liquid extraction (PLE) techniques using response surface methodology to optimize phenolic compound yield and antioxidant capacity. Specifically, a D-optimal design was applied for EAE, and a Box–Behnken design was applied for PLE. The optimal extraction conditions for EAE were 0.75 U/mL of tannase, 40 U/mL of cellulase, 20 °C, and 15 min. For PLE, the optimal parameters were 54% ethanol, 113 °C, and three extraction cycles. These conditions yielded 38.49 mg GAE g^−1^ dw and 50.03 mg GAE g^−1^ dw of total polyphenols and antioxidant capacities of 342.47 μmol TE g^−1^ dw and 371.00 μmol TE g^−1^ dw, respectively. The extracts obtained under optimal conditions were further characterized through chromatographic techniques to determine their phenolic profiles. Seven phenolic compounds were identified: gallic acid, catechin, epicatechin, 4-hydroxybenzoic acid, quercetin-3-rutinoside hydrate, quercetin-3-O-rhamnoside, and kaempferol. PLE extracts exhibited the highest concentration of these compounds. These findings demonstrate that recovering antioxidant-rich phenolic compounds from pisco grape pomace using innovative extraction methods is a viable strategy for obtaining functional ingredients and supporting sustainable industrial practices.

## 1. Introduction

Grapes are one of the most produced fruits and are essential in the global economy, with a production of approximately 75 million tons/year. Their production centers on fresh consumption, juices, raisins, wine, and derivatives [1,2]. In Chile, pisco is a distilled beverage obtained from grapes, and this significant industry is concentrated in the Atacama and Coquimbo regions, with the majority of grape plantations located in the latter region [3]. The pisco process generates grape pomace, a by-product primarily composed of seeds and skins, which accounts for approximately 20–30% of the weight of harvested grapes [4]. In the Coquimbo region, around 6000 tons of grape pomace are generated annually. It is currently used for composting and as free animal feed, yet this disposal method continues to impact soil and groundwater negatively [4]. Due to its composition, grape pomace is rich in polyphenols, such as gallic acid, catechin, and quercetin (in concentrations between 400 and 800 ug/g), along with other bioactive secondary metabolites [4,5]. Interest in these compounds is growing, as they have demonstrated various health-promoting properties and potential in disease prevention [2,6]. The solution to the problem of the final disposal of this pomace is to find an appropriate technology for valorization so that it can be transformed into a product with added value for marketing.

Conventional extraction (solid–liquid) is generally used to recover polyphenols from different plant matrices. However, this process has several drawbacks, as it uses various toxic solvents in high quantities, generating environmental risk, long extraction processes, and low yields [7]. Among the non-conventional methods is enzyme-assisted extraction (EAE), a more environmentally friendly alternative strategy based on enzymes’ ability to degrade complex cell wall materials, thus promoting the release of trapped compounds [8]. The cell wall of grape skins is a complex network formed by various components such as pectin, lignin, proteins, and polysaccharides. The phenols in grapes include those that bind to these latter constituents through hydrogen bonds and hydrophobic interactions [9], and enzymes could release them [10]. The action of cellulases consists of the depolymerization of cellulose into simpler fermentable sugars, and tannase hydrolyzes the ester and depside bonds of tannins, producing glucose and gallic acid. Therefore, a selective enrichment in different extract phenols is expected depending on the action of each enzyme [9]. Another type of extraction is pressurized liquid extraction (PLE), which has several advantages over other extraction methods. Among these is its mechanism that establishes that high temperatures and pressure promote greater recovery of the phenolic compounds in a short period because there is an increase in the mass transfer rate from the matrix to the solvent [11]. However, at temperatures above 120 °C, these bioactive compounds undergo degradation, which is considered an operating range for phenolic compound extraction [12]. The pisco grape pomace extraction has been little studied; to date, technologies such as ultrasound and Hot Pressurized Liquid have been utilized, providing recovery alternatives [5,7].

Emerging extraction techniques such as enzyme-assisted extraction (EAE) and pressurized liquid extraction (PLE) are gaining increasing attention as environmentally friendly and sustainable strategies for recovering bioactive compounds from agricultural by-products. Response surface methodology (RSM) offers a valuable set of statistical and mathematical tools to develop, optimize, and improve these extraction processes. RSM enables the evaluation of the effects of independent variables on response variables through experimental designs that reduce the number of required trials. Standard designs used for optimizing the extraction of polyphenolic compounds include Central Composite Design (CCD), Box–Behnken, and D-optimal designs, among others [13,14].

The main objective of this study is to use two emerging extraction technologies (EAE and PLE) for the recovery of phenolic compounds and antioxidants from Pisco grape pomace, applying experimental design models to find the best conditions in both extractions to obtain compounds with health benefits and thus use and valorize this plant residue as a potential source for the future development of functional foods.

## 2. Results and Discussion

### 2.1. Characterization of the Dried Pomace

Table 1 shows a proximate analysis of the vacuum-dried sample. The moisture content of the final product was 5.51 g per 100 g^−1^, expressed on a dry basis and an a_w_ of 0.3069. The water activity must be less than 0.6 to ensure that the raw matrix is microbiologically stable, thus increasing its shelf life [4]. The lipid content was 7.35 g per 100 g^−1^ dw, mainly attributed to grape pomace seeds [15]. The ashes and proteins presented values of 5.83 and 12.74 g 100 g^−1^ dw, respectively. These values of the proximate analysis varied with respect to those of other studies on grape pomace in which the following values were obtained: moisture (1.59–9.77 g per 100 g^−1^), lipids (2.12–13.5 g per 100 g^−1^ ), ashes (2.4–23.7 g per 100 g^−1^), proteins (7.52–13.9 g per 100 g^−1^), sugars (2.1–14.2 g per 100 g^−1^), and carbohydrates (77–79.11 g per 100 g^−1^) [5,16,17,18].

The total dietary fiber (TDF) content was high, at 40.94%. It should be noted that the total dietary fiber is composed of lignins, celluloses, and hemicelluloses that are linked to insoluble dietary fiber (38.08%), the predominant fraction present in the pomace. The literature shows that the TDF in grape varieties is 35–55% [15], while in the pomace in red grape varieties, a content of 16.4–58% TDF was found [19].

The total carbohydrate content was 74.07 g per 100 g^−1^ dw, with reducing sugars expressed as glucose accounting for 33.65 g per 100 g^−1^ dw. This indicates a substantial amount of simple sugars, with the remaining fraction mainly corresponding to dietary fiber. These findings are in line with those reported for pisco grape pomace dehydrated using different drying methods and similar grape varieties [16]. In pomace, carbohydrates can vary between 6 and 15% depending on the grape variety, agro–climatic conditions, or viticultural practices. It should be considered that these white pomaces contain a relatively high number of soluble sugars, unlike pomace from red grapes [5].

Taken together, the compositional profile characterized by high fiber, relevant sugar content, and low water activity supports not only the stability and conservation of the dried matrix but also its potential as an optimal substrate for emerging extraction technologies, aimed at the efficient recovery and valorization of its bioactive compounds.

### 2.2. Experimental Design of Enzyme-Assisted Extraction

To define the experimental design, preliminary tests were carried out to determine the factors and levels to be used. The effect of three enzymes, pectinase, cellulase, and tannase—applied individually at different concentrations—on the extraction yield of total polyphenols was evaluated. According to the results, cellulase and tannase increased polyphenol extraction in a concentration-dependent manner, showing a progressive improvement in yield with increasing dosage. In contrast, pectinase showed no significant improvement in polyphenol recovery, irrespective of the enzyme concentration used. This result can be explained by the low pectin content present in the pomace (3.02%), as reported by Vásquez et al. [16]. Several studies have shown a positive effect of using individual and combined enzymes [9]. The action of cellulase is because its substrate is cellulose, one of the polysaccharides found in the highest proportion in the pomace (12%) and is characterized by giving stability and rigidity to the cell wall. Therefore, its degradation allows access to the compounds trapped in its structure, and tannase can depolymerize the polyphenols present [8].

D-optimal experimental design using response surface methodology (RSM) was performed to establish the optimal conditions for maximizing total polyphenol extraction yield (TPC) and antioxidant capacity (DPPH). The effects of four independent variables, tannase (U/mL), cellulase (U/mL), temperature (°C), and time (min), were investigated. Table 2 shows the experimental values of the 27 runs of the design for each of the response variables evaluated.

The most reliable way to assess the quality of the adapted model is to apply the analysis of variance (ANOVA), which consists of determining the effects of several independent variables on the response variable and deciding whether these effects are significant [20].

According to the ANOVA (Table 3) analysis for TPC, the value of R^2^ = 0.9639, and the adjusted R^2^ = 0.9456. The R^2^ is related to the terms of the mathematical model, while the adjusted R^2^ is the one that allows for making decisions and relates to the quality of the fit; it also indicates that the model obtained can explain 94.56% of the variation in the TPC value [14]. The coefficient of variation (CV) is a relationship between the mean and the standard deviation. A low CV value indicates that there is a high reproducibility of the results obtained [5]; for this model, a CV value of 6.32% was obtained.

An analysis of the quadratic regression model showed that it was significant (*p* < 0.05), while the “lack of fit” was not significant (*p* > 0.05). Of the four independent variables in the model, temperature and time were significant (*p* < 0.05) at the 95.0% confidence level. The model showed that the independent variable with the most significant effect on the TPC was time (X_4_), with a lower *p*-value (*p* < 0.05) and an F value of 218.54, followed by temperature (X_3_), with an F value of 78.81. At the same time, tannase (X_1_) and cellulase (X_2_) did not significantly affect the TPC yield (*p* > 0.05). A reduced quadratic model was developed since the quadratic independent variables and interactions that did not present significance in the design for the TPC data set were eliminated in the analysis and adjusted to the following Equation (1):

(1) $$TPC\left(Y_{1}\right)=20.51+{0.3892X}_{1}+{0.9420X}_{2}-{3.86X}_{3}-{6.71X}_{4}-{2.66X}_{3}X_{4}+{2.00X}_{1}^{2}+{8.46X}_{3}^{2}+{2.09X}_{4}^{2}$$

For the antioxidant capacity, the value of R^2^ = 0.8698 and the adjusted R^2^ = 0.8116 presented a % CV of 6.13%. An analysis of the quadratic regression model showed that it was significant (*p* < 0.05), while the “lack of fit” was not significant (*p* > 0.05). Of the four independent variables in the model, temperature (X_3_) and time (X_4_) were significant again (*p* < 0.05) at the 95.0% confidence level. The model showed that the independent variable with the greatest effect on the extraction yield was time (*p* < 0.05), with an F value of 44.46, followed by temperature, with an F value of 28.05. Meanwhile, tannase and cellulase did not have significant effects on the antioxidant yield (*p* > 0.05) (Table 3).

The quadratic model developed in the antioxidant capacity data set was adjusted to the following Equation (2):

(2) $$Antioxidant\left(Y_{2}\right)=257.25-6.52X_{1}+{5.00X}_{2}-{21.75X}_{3}-{27.49X}_{4}-{15.74X}_{1}X_{2}-{10.83X}_{3}X_{4}+{25.65X}_{3}^{2}+{22.05X}_{4}^{2}$$

**Figure 1:**
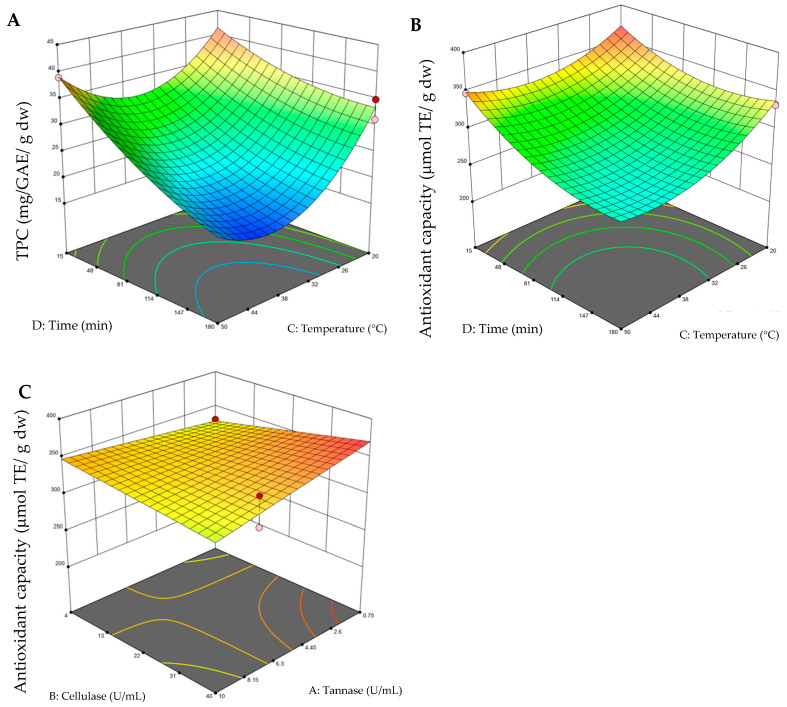
Three-dimensional surface graphs from enzyme-assisted extraction (EAE). (**A**) Total polyphenol content (TPC) (time–temperature); (**B**) antioxidant capacity (time–temperature); (**C**) antioxidant capacity (tannase–cellulase). On the color scale: blue represents low values and red high values.

The 3D surface graphs were obtained by keeping two variables constant and varying the other two within the experimental range. Figure 1 depicts the impacts of EAE conditions, such as enzyme type, temperature, and time, on TPC and antioxidant capacity. Figure 1A,B shows that a shorter extraction time (15 min) and a lower temperature (20 °C) result in a higher TPC yield and greater antioxidant capacity (with fixed variables of 40 U/mL of cellulose and 0.75 U/mL of tannase, for viewing the grap), but it is also observed at a high temperature (50 °C). It can be observed that the highest values of the response variable were found at the extremes of the experimental levels; this can be attributed to the fact that the range of levels chosen was limited for this type of extraction. Considering this, additional experimental tests were carried out, where temperatures of 10 °C and 60 °C were evaluated, as well as times of 10 min and 200 min; here, it was confirmed in these ranges that both the content of total polyphenols (21.89–34.38 mg GAE g^−1^ dw) and antioxidant capacity decreased (221.47–264.64 μmol ET g^−1^ dw). As mentioned above, the TPC 3D surface graphs evaluating the effect of enzymes (tannase–cellulase) were not shown because it was not significant for the model. Figure 1C shows that the combination of a higher amount of cellulase (40 U/mL) and a lower amount of tannase (0.75 U/mL) results in an improved antioxidant capacity, maintaining a temperature and time of 20 °C and 15 min, respectively. In addition, it was found that the TPC value varied in wide ranges from 16.19 to 44.78 mg GAE g^−1^ dw depending on the changes in the process variables, while the antioxidant capacity varied from 226.34 to 369.49 μmol ET g^−1^ dw (Table 2). The optimal conditions of EAE to maximize the TPC and antioxidant capacity were 0.75 U/mL of tannase, 40 U/mL of cellulase, 20 °C, and 15 min, with a desirability value of 0.734, where values close to 1 indicate that the response value is consistent with the target value.

### 2.3. Experimental Design of Pressurized Liquid Extraction (PLE)

According to preliminary experiments and a literature search based on studies that have used PLE to extract different compounds from grapes [12,21,22], the experimental design was defined considering the effects of three independent variables: ethanol concentration, temperature, and extraction cycles, evaluating the phenolic content and the antioxidant capacity. Table 4 shows the experimental values of the 17 design runs for each of the response variables evaluated.

A quadratic model was developed for the total polyphenol and antioxidant capacity data set. In the ANOVA analysis for TPC, the value of R^2^ = 0.9889, the adjusted R^2^ = 0.9747, and the CV (%) was 6.37 (Table 5). A quadratic model was developed, considering the interactions and quadratic independent variables, and the analysis was adjusted to the following Equation (3):

(3) $$TPC\left(Y_{3}\right)=43.27+11.17X_{1}+7.40X_{2}+0.8588X_{3}-3.39X_{1}X_{2}-1.12X_{1}X_{3}-0.07X_{2}X_{3}-17.81{X_{1}}^{2}\;\;+0.8715{X_{2}}^{2}-3.20{X_{3}}^{2}$$

The antioxidant capacity, the value of R^2^ = 0.9777, and the adjusted R^2^ = 0.9490 presented a CV (%) of 8.82. The quadratic model developed in the antioxidant capacity data set was adjusted to the following Equation (4):

(4) $$Antioxidant\left(Y_{4}\right)=298.81+63.69X_{1}+24.59X_{2}+1.86X_{3}-29.91X_{1}X_{2}-8.01X_{1}X_{3}-21.08X_{2}X_{3}-98.51{X_{1}}^{2}\;\;+25.27{X_{2}}^{2}-16.16{X_{3}}^{2}$$

An analysis of the quadratic regression model showed that it was significant (*p* < 0.05), while the “lack of fit” was not significant (*p* > 0.05). The model showed that the independent variables with the most significant effect on TPC yield were ethanol concentration (X_1_), with an F value of 215.09, and temperature (X_2_), with an F value of 94.47 (*p* < 0.05), while for antioxidant capacity, the independent variable with the most significant effect was ethanol concentration (X_1_) (*p* < 0.05), with an F value of 114.05. The extraction cycle variable (X_3_) for both response variables was not significant (*p* > 0.05).

**Figure 2:**
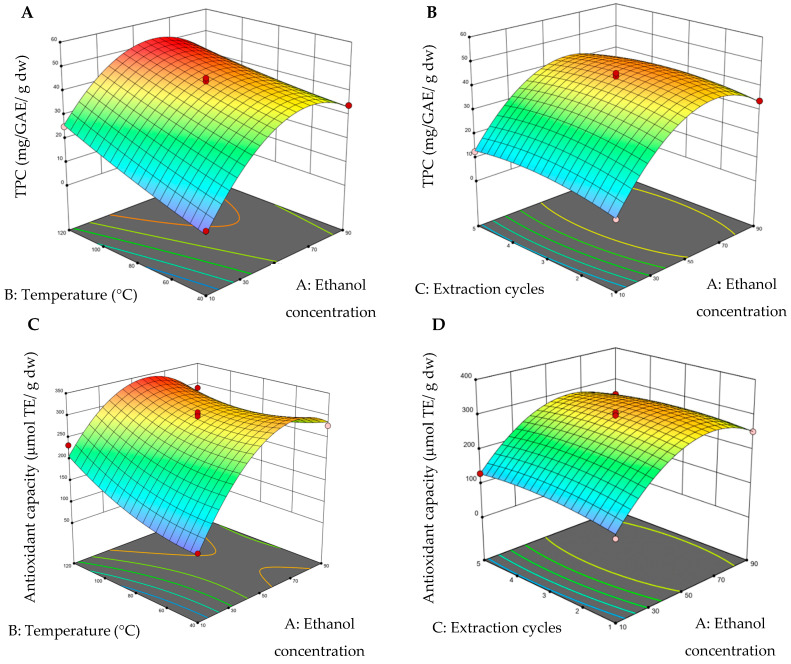
Three-dimensional surface graphs from pressurized liquid extraction (PLE). (**A**) Total polyphenol content (TPC) (ethanol concentration–temperature); (**B**) total polyphenol content (TPC) (ethanol concentration–extraction cycles); (**C**) antioxidant capacity (ethanol concentration–temperature), and (**D**) antioxidant capacity (ethanol concentration–extraction cycles). On the color scale: blue represents low values and red high values.

The 3D surface graphs were obtained by keeping one variable constant and varying the other two within the experimental range. Figure 2 shows the impacts of PLE conditions, such as ethanol concentration, temperature, and extraction cycles, on TPC and antioxidant capacity.

Figure 2A,C shows that the yield of total phenolic compounds (TPCs) and antioxidant capacity increase significantly when using temperatures between 80 and 120 °C and ethanol concentrations between 50 and 80%, with three extraction cycles kept constant. In other studies temperature (such as 90–150°) and solvent concentration (such as 30–70%) ranges are consistent with our study in which similar conditions enhanced the extraction efficiency of phenolic compounds, attributed to improved solubility and greater release of phenolic metabolites from the plant matrix [23,24,25]. It is observed that the red regions in Figure 2B,D exhibit a tendency similar to the maximum zone of the response variable, concerning the extraction cycles, and in the ethanol concentration region (with a fixed variable of 80 °C for viewing the graph). In addition, it was found that the TPC value varied in wide ranges from 5.84 to 50.21 mg GAE g^−1^ dw, while the antioxidant capacity varied from 98.33 to 347.14 μmol ET g^−1^ dw (Table 4). Some studies in PLE have reported that extraction cycles did not show significance in obtaining TPC, using longer extraction times (10–20 min) [21,22].

The optimal conditions for extracting total polyphenols and antioxidant capacity for PLE were 54% ethanol, 113 °C, and three extraction cycles with a desirability value of 0.937, where values close to 1 indicate that the response value is consistent with the target value.

### 2.4. Validation of Enzymatic and Pressurized Liquid Extractions

To verify the reliability of the models of both extractions, experiments were carried out under optimal conditions, in which the experimental values were compared with the predicted values to obtain the percentage error (Table 6). The percentage errors for EAE were 7.34% and 7.57% for TPC content and antioxidant capacity, respectively. In the case of PLE, they were 1.24% and 11.80% for TPC and antioxidant capacity, respectively. It is important to mention that these variations can be attributed to the samples’ heterogeneity due to their composition (seeds and skin). The errors between the predicted and experimental values indicate that through extrapolation, the regression models obtained by RSM could predict the extraction yields of TPC and antioxidant capacity.

### 2.5. Characterization of Optimal Extracts

Table 7 presents the characterization of the optimal extracts obtained by EAE and PLE. The content of the total polyphenols, total flavonoids, antioxidant capacity (using DPPH and ORAC assays), sugars, and phenol profile was evaluated.

The optimized extracts presented higher amounts of phenolic compounds and antioxidant capacity than by agitation. Compared with conventional extraction (CE), the EAE extract increased 31% TPC, and increased antioxidant capacity by 2% and 8% using DPPH and ORAC, respectively. Meanwhile, PLE stood out over agitation extraction in that the PLE extract increased 70% TPC, and increased antioxidant capacity by 12% and 24% using DPPH and ORAC, respectively. Both extracts showed significant differences between them (*p* < 0.05).

CE showed a polyphenol content of 29.46 ± 0.37 mg GAE g^−1^ dw, flavonoid content of 48.04 ± 0.75 mg QE g^−1^ dw, and antioxidant capacity values of 329.67 ± 5.39 and 1549.17 ± 94.63 μmol ET g^−1^ dw using DPPH and ORAC, respectively.

The sugars in EAE and PLE increased by 20% compared to CE (217.03 mg glucose g^−1^ dw). The sugar content was determined considering that grape skin contains large amounts of hemicellulosic sugars, which increase when hydrolyzed due to the release of xylose and glucose monomers [19].

To evaluate the distribution of polyphenolic compounds within the different fractions of grape pomace, the optimal extraction conditions obtained in this study were applied separately to the seed and skin components. EAE in seeds had a TPC content of 58.48 mg GAE g^−1^ dw, while the skin had 8.24 mg GAE g^−1^ dw. The values for antioxidant capacity with DPPH were 401.30 μmol ET g^−1^ dw and 171.86 μmol ET g^−1^ dw for the seeds and skin, respectively. For PLE, the TPC content was 67.73 mg GAE g^−1^ dw in seeds and 22.44 mg GAE g^−1^ dw in the skin. The values for antioxidant capacity using DPPH were 399.21 μmol ET g^−1^ dw in seeds and 171.86 μmol ET g^−1^ dw in the skin. Notably, in all grape varieties, the highest concentration of polyphenolic compounds is present in the seeds, which are correlated with the antioxidant capacity. In addition, 70% of the extractable compounds are from the seeds, while 28–35% are from the skin [15]. The TPC values obtained in this work were higher than those reported in other grape extraction studies. Other research studies using EAE reported values ranging from 0.1 to 20 mg GAE/g, while PLE studies showed ranges of 0.46–15.24 mg GAE/g. EAE variations can be attributed to the type of grape (red and white), type of enzyme, the enzyme–substrate ratio, longer incubation times, and higher temperatures [7,26,27]. In PLE, the variations can be attributed to the type of grape *(Quebranta* and *Torontel*), higher temperature, and longer extraction times [7,21].

EAE and PLE, compared to ultrasonic extraction (15 min, 70% amplitude, 65 °C, and 20 kHz) and microwave extraction (2.45 Ghz, 10 min, and 73 °C), which are also emerging techniques applied to pomace and the seeds of red and white grape varieties, obtained similar values in TPC (44.56–81.13 mg GAE g^−1^) and lower values in antioxidant capacity using DPPH (27.56–77.64 μmol ET g^−1^) [28]. Although studies have been conducted on optimizing EAE and PLE in grape pomace for wine production, work has yet to be reported using enzymes in pisco grape pomace. In this case, PLE proves to be more efficient in increasing the content of the compounds of interest because this technique operates at high temperatures and pressures below critical points of solvent (maintained in the liquid state during whole extraction procedure), enhancing mass transfer, reducing viscosity, and increasing solubility, which allows for deeper matrix penetration and higher extraction yields than the conventional method [29].

The correlation between TPC and antioxidant capacity was evaluated for the EAE and PLE extracts. The EAE extract showed R^2^ = 0.8017, and the PLE extract showed R^2^ = 0.8827; these values indicate a high correlation between both variables studied (Supplementary Materials).

### 2.6. Phenol Profile of Optimum Extracts

Phenolic compounds are natural antioxidants that control or prevent metabolic syndrome and various chronic diseases. They also possess various pharmacological activities, such as antimicrobial, anti-inflammatory, and anticarcinogenic properties [19,21,30].

**Figure 3:**
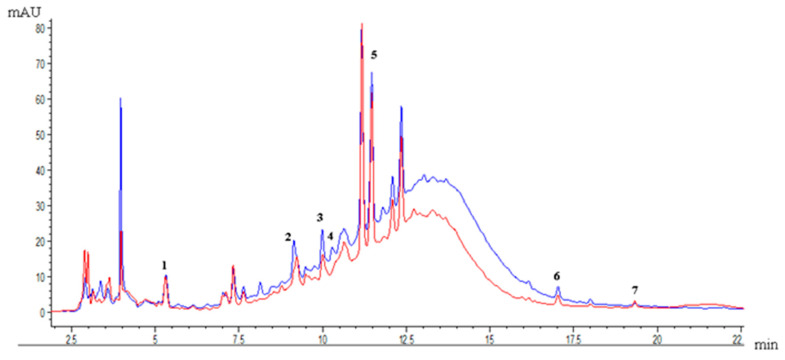
Profile of phenolic compounds from pisco grape pomace: (1) gallic acid, (2) catechin, (3) epicatechin, (4) 4-hydroxybenzoic acid, (5) quercetin-3-rutinoside hydrate, (6) quercetin-3-O-rhamnoside, (7) kaempferol. Comparison of the profile of phenolic compounds of pisco grape pomace enzyme-assisted extraction (red) and pressurized liquid extraction (blue).

Figure 3 compares the profile of phenolic compounds present in the optimal EAE and PLE extracts. The phenolic compounds identified in both optimum extractions were gallic acid, catechin, epicatechin, 4-hydroxybenzoic acid, quercetin-3-rutinoside hydrate, quercetin-3-O-rhamnoside, and kaempferol.

The PLE extract showed the highest concentration of phenolic compounds compared to EAE. Quercetin-3-rutinoside hydrate was the compound in the highest concentration, with a value of approximately 3 mg g^−1^ dw (Table 7). It was followed by catechin and epicatechin, with values of 0.3–0.7 mg g^−1^ dw, respectively. Due to the advantages of PLE already mentioned, and the added thermal energy, this extraction contributes to breaking the matrix bonds and favors the diffusion of some specific polyphenols, such as phenolic acids, flavanols, and flavonols [7].

EAE and PLE demonstrated superior phenolic recovery compared to EA from grape pomace extracts. Chromatographic analysis (HPLC–DAD) revealed that both EAE and PLE extracted a greater number of individual phenolic compounds than EA. According to a previous study by Poblete et al. [4], which detailed the EA chromatogram, a comparison with optimized EAE and PLE extracts revealed that these advanced extraction methods not only increased the concentration of existing compounds but also enabled the identification of two additional phenolic compounds: quercetin-3-O-rhamnoside (0.09–0.12 mg g^−1^ dw) and kaempferol (0.04–0.05 mg g^−1^ dw), which were also identified and quantified. Most of the compounds found belong to the flavonol group.

Under optimal conditions, individual phenolic compounds were also evaluated in the seeds and skins of grape pomace. The EAE method proved to be more effective in recovering catechin and epicatechin from seeds, yielding concentrations of 0.45–1.45 mg g^−1^ dw compared to 0.36–1.26 mg g^−1^ dw obtained with PLE. Separating the fractions (skin and seeds) significantly increases the exposed surface area, allowing the enzymes to more easily access the substrates and improving their specificity. Thus, the tannins (or proanthocyanidins) of seeds are hydrolyzed, and their main monomers are obtained, such as catechin, epicatechin, epigallocatechin, and epicatechin-3-O-gallate [31]. On the other hand, the skin had a higher concentration of quercetin-3-rutinoside hydrate (2.59 mg g^−1^ dw) using EAE.

Some studies on grapes and grape pomace using EAE and PLE reported the presence of gallic acid, catechin, epicatechin, caffeic acid, rutin, quercetin, and kaempferol. These studies reported that gallic acid was one of the predominant compounds; rutin and quercetin were found in higher concentrations, while catechin and epicatechin were found in lower concentrations, mainly in white grapes [7,32].

Among the bioactive properties of the phenolic compounds found, gallic acid (0.02–0.253 mg g^−1^ dw) is one of the most important hydroxybenzoic acids found in grapes; it can be found in the skin, pulp, or seeds but is more abundant in the skin, and it has a high antioxidant capacity. Catechins (0.04–0.09 mg g^−1^ dw) and epicatechins (0.01–0.04 mg g^−1^ dw) have cholesterol-lowering properties and reduce blood pressure. Rutin (0.421 mg g^−1^ dw), quercetin (0.012 mg g^−1^ dw), and kaempferol (0.005 mg g^−1^ dw) possess potent antioxidant capacity, playing a role in protecting against cardiovascular disease. These last two compounds, belonging to the flavonols group, are mainly present in grapes and grape pomace as aglycones and in glycosylated form [7,19,32,33].

## 3. Materials and Methods

### 3.1. Materials

The pisco grape pomace was obtained from Pisquera de Chile S.A., located in Ovalle, Coquimbo Region, Chile, during the 2021 harvest, corresponding to the Muscat variety (Muscat Rosada (60%) and Muscat Alejandria (40%)), with an initial moisture content of 47%.

### 3.2. Chemicals and Reagents

The enzymes used were *Aspergillus niger* cellulase (white powder, ~0.8 U/mg) (Sigma-Aldrich, St. Louis, MO, USA) and food-grade tannase (white powder ≥ 100 U/g) (high-tech industrial zone, Xi´an, China).

Ethanol, water milli-Q, Folin–Ciocalteu reagent, sodium carbonate, DPPH (2,2-Diphenyl-1-picrylhydrazyl), AAPH (2,2′-Azobis (2-methylpropionamidine dihydrochloride), fluorescein sodium salt, Trolox (6-hydroxy-2,5,7,8-tetramethylchroman-2-carboxylic acid), acetonitrile, water (HPLC grade), and formic acid were purchased from Merck (Darmstadt, Germany). Glucose 3,5-dinitrosalicylic acid and standards of the phenolic compounds gallic acid, catechin, epicatechin, 4-hydroxybenzoic acid, quercetin-3-rutinoside hydrate, quercetin-3-O-rhamnoside, and kaempferol were purchased from Sigma Aldrich Co. (St. Louis, MO, USA).

### 3.3. Preparation of the Raw Material

Once the grape pomace was received, it was dehydrated in a vacuum oven at a temperature of 60 °C with a pressure of 100 mbar (Memmert, model VO 400, Schwabach, Germany) for 390 min based on a study carried out in a previous work [4]. The sample was stored until further analysis at −18 °C.

### 3.4. Characterization of the Physico-Chemistry of the Dried Pomace

The proximate analysis of dried grape pomace was measured according to the methodology of the Association of Official Analytical Chemists [34]. The analysis included the determination of moisture content, lipid content, ash content, protein content, and dietary fiber content, and the carbohydrates were calculated by difference. Water activity (a_w_) was measured at 25 °C using AQUA LAB equipment (4TE, Pullman, WA, USA). Reducing sugar content was measured according to Bailey et al. [35] and expressed as grams of glucose equivalent to 100 g^−1^ dw. All measurements were performed in triplicate.

### 3.5. Conventional Extraction by Agitation (CE)

Conventional extraction was performed following the methodology described by Li et al. [21] with minor modifications. One g of sample was weighed and mixed with 40 mL of 50% ethanol for 30 min in an orbital shaker (Boeco, OS-20) at 200 rpm. It was then centrifuged for 5 min at 4193× *g*, and the supernatant was filtered for further analysis.

### 3.6. Experimental Design and Extraction of Enzyme-Assisted Extraction (EAE)

A D-optimal design was used to optimize the EAE parameters and evaluate the main influences of various parameters on the total polyphenol extraction yield (Y_1_) and antioxidant capacity (Y_2_) of grape pomace. Four independent variables (tannase (X_1_), cellulase (X_2_), temperature (X_3_), and time (X_4_)) with their levels (0.75–10 U/mL; 4–40 U/mL, 20–50 °C and 15–180 min, respectively) were considered, and the response variables were evaluated to determine the optimal extraction condition. These enzyme concentration ranges (U/mL) were selected based on preliminary studies that evaluated the enzyme–substrate (pomace) relationship, selecting a fixed mass of pomace of 1 g to establish optimal enzymatic conditions. The experimental data obtained were fitted to a second-order polynomial regression model. For the extraction procedure, one g of dry pomace was weighed and mixed with 20 mL of acetate buffer (0.1 M, pH 4.8), and the dose of each enzyme was added and subjected to the process conditions delivered by the design [7]. Then, the sample obtained in EAE was taken to a conventional extraction by shaking under the procedure described by Li et al. [21] to separate the released compounds. Then, 20 mL of absolute ethanol was added to the previous mixture and shaken for 30 min (Boeco, OS-20). It was centrifuged, filtered, and reconstituted to a known volume.

### 3.7. Experimental Design and Extraction of Pressurized Liquid Extraction (PLE)

A Box–Behnken design was used to optimize the PLE parameters and evaluate the main influences of various parameters on the TPC extraction yield (Y_3_) and antioxidant capacity (Y_4_) of grape pomace. Three independent variables (ethanol concentration (X_1_), temperature (X_2_), and extraction cycles (X_3_) with levels of 10–90% (ethanol: water), 40–120 °C and 1–5 cycles, respectively) were considered, and the response variables were evaluated to determine the optimum extraction condition. The experimental data obtained were fitted to a second-order polynomial regression model. For the extraction procedure, one g of dry pomace was weighed and mixed with diatomaceous earth (1:1). The solvent (X_1_) was circulated through a 34 mL stainless steel cell and taken for accelerated solvent extraction (Thermo Scientific Thermo Fisher Scientific Dionex ASE 150, EE.UU) according to the experimental design. The fixed conditions of the process were 3 min of extraction (according to the preliminary assay, the time of extraction from 3 to 15 min was evaluated) and 103 bar of pressure. These conditions were selected according to the total polyphenol yield performance. Then, the supernatant was centrifuged, filtered, and reconstituted to a known volume [21].

### 3.8. Characterization of Optimal EAE and PLE Extracts

Total phenolic compound (TPC) content was assessed using a Folin–Ciocalteu assay based on the Singleton and Rossi method [36] in a multiplate reader (Perkin-Elmer, Victor TM χ3, Turku, Finland) at 750 nm using gallic acid as the standard (0.02–1.00 mg/mL). Antioxidant capacity determination using a DPPH assay was performed using the method described by Grajeda-Iglesias et al. [37] in a multiplate reader at 510 nm. Total flavonoid content (TPC) was determined in a spectrophotometer (Thermo Scientific, ORION AQUAMATE 8000, Madison, WI, USA) at 415 nm using quercetin as the standard (0.25–0.5 mg/mL) [38]. The ORAC assay was performed in a 96-well microplate at an excitation wavelength of 485 nm and an emission wavelength of 535 nm [38]. Trolox was used as the standard in all antioxidant assays (1–500 μM). Reducing sugar content was determined using dinitrosalicylic acid reagent (DNS) according to the method of Bailey et al. [35] using glucose as the standard (0.1–3.0 mg/mL). For the determination of the phenolic profile, the extracts were automatically injected into an HPLC system (Agilent 1200 series, Santa Clara, CA, USA) with a diode array detector (DAD). The HPLC system was equipped with a Kromasil 100-5C18 column (250 × 4.6 mm; Eka Chemical, Bohus, Sweden) with a spherical particle size of 5 μm, maintained at 25 °C with a flow rate of 0.7 mL min^-1^. The injection volume was 10 μL, and the mobile phase comprised 0.1% formic acid, pH 3 (A), and 100% acetonitrile (B). The elution gradient was initially set at 87% A and 13% B from 0 to 16 min, 45% A and 55% B from 16 to 23 min, 60% A and 40% B between minutes 23 and 25, and 87% A and 13% B between minutes 25 and 30, and then returned to initial conditions after 4 min. The absorption spectra of the main peaks were recorded at 280, 310, and 370 nm. For quantification, calibration curves were performed for gallic acid (1–100 ppm), catechin (10–300 ppm), epicatechin (10–100 ppm), 4-hydroxybenzoic acid (10–100 ppm), quercetin-3-rutinoside hydrate (10–100 ppm), quercetin-3-O-rhamnoside (1–80 ppm), and kaempferol (1–40 ppm) [38].

### 3.9. Statistical Analysis

The selected D-optimal design consisted of 27 experiments, which included four central points for calculating variance, and the Box–Behnken design consisted of 17 experimental runs. For both designs, statistical regression validation was performed using ANOVA with a confidence level of 95%. The optimal conditions of the variables that maximize the response were calculated with RSM using the Design-Expert software (test version 8.0.6, Stat Ease Inc., Minneapolis, MN, USA). In addition, the effect of both extractions was evaluated using an analysis of variance and the multiple-range test. Statistically significant differences were determined when the *p*-value was <0.05 (Statistical Graphics Corp., Herndon, VA, USA).

## 4. Conclusions

The experimental design of the emerging extraction processes presented in this work allowed for an increase in the extraction yield of bioactive compounds such as polyphenols and flavonoids with antioxidant properties compared to a conventional shaking technique. The optimal condition of EAE was 0.75 U/mL tannase, 40 U/mL cellulose, 20 °C, and 15 min. Compared with CE, EAE increased the polyphenol yield and antioxidant capacity by 31% and 3%, respectively. The optimal conditions of PLE were 54% ethanol, 113 °C, and three extraction cycles, increasing the polyphenol yield and antioxidant capacity by 70% and 3%, respectively, compared to CE. PLE showed the highest total polyphenol content and antioxidant capacity among both optimal extracts. Seven phenolic compounds were found in EAE and PLE: gallic acid, catechin, epicatechin, 4-hydroxybenzoic acid, quercetin-3-rutinoside hydrate, quercetin-3-O-rhamnoside, and kaempferol, with rutin predominating, followed by catechin and epicatechin. The phenolic compounds found in grape pomace from pisco production extracted by emerging techniques are the first step in their consideration to be an alternative in high-value-added products, such as new food ingredients, nutraceuticals, sustainable packaging, and others, contributing to sustainability and generating a promising solution to develop products with benefits for human health.

## Figures and Tables

**Table 1:** Proximal composition of vacuum-dried (VAC) grape pomace at 60 °C.

Parameters	VAC 60 °C
^1^ Moisture	5.51 ± 0.26
^2^ Fat	7.35 ± 0.24
^2^ Ash	5.83 ± 0.09
^2^ Crude protein	12.74 ± 0.54
^2^ Insoluble dietary fiber, IDF	38.08 ± 4.04
^2^ Soluble dietary fiber, SDF	2.86 ± 0.18
^2^ Total dietary fiber, TDF	40.94 ± 3.86
^3^ Total carbohydrates	74.07 ± 0.48
^4^ Reducing sugar content	33.65 ± 1.09
^5^ Water activity, a_w_	0.3069 ± 0.0066

^1^ Mean value ± standard deviation (SD) was expressed in g 100 g ^−1^ sample (%). ^2^ Mean values ± SD and the data are expressed as g 100 g^−1^ dw. ^3^ Total carbohydrates (TCs) by difference: TC = 100 − (% moisture + % crude protein + % fat + % ash). ^4^ Reducing sugar content is expressed as g glucose 100 g^−1^ dw. ^5^ Dimensionless.

**Table 2:** Experimental D-optimal design for EAE of pisco grape pomace yield of extract polyphenols and the antioxidant capacity.

	Independent Variables						Response Variables	
	X_1_	X_2_	X_3_	X_4_	Y_1_		Y_2_	
Run	Tannase	Cellulase	Temperature	Time	TPC		Antioxidant Capacity by DPPH	
	(U/mL)	(U/mL)	(°C)	(min)	(mg GAE g^−1^ dw)		(μmol TE g^−1^ dw)	
					Predicted	Experimental	Predicted	Experimental
1	3.99	4	50	15	35.69	35.61 ± 0.16	313.76	327.04 ± 2.70
2	0.75	40	20	180	33.42	31.17 ± 0.05	337.28	332.94 ± 9.92
3	0.75	4	20	15	39.63	38.96 ± 1.20	329.15	300.68 ± 6.47
4	10	4	20	88.43	34.74	33.80 ± 0.79	310.97	329.88 ± 7.96
5	10	4	50	180	19.27	19.47 ± 0.06	249.10	260.06 ± 1.87
6	5.38	22	35	97.5	20.51	20.28 ± 1.09	257.25	253.04 ± 2.22
7	3.99	4	20	180	29.99	31.68 ± 0.54	302.27	320.17 ± 7.48
8	10	40	33.05	180	20.21	23.23 ± 3.18	239.21	252.69 ± 1.29
9	10	40	50	88.43	29.49	30.21 ± 1.56	248.38	258.26 ± 8.25
10	10	24.16	20	180	33.37	30.11 ± 2.37	302.22	289.96 ± 14.51
11	7.6	40	20	15	40.55	40.09 ± 0.87	337.68	320.78 ± 10.58
12	0.75	40	20	180	33.42	34.92 ± 0.65	337.28	331.81 ± 5.45
13	0.75	4	20	15	39.63	39.79 ± 0.32	329.15	331.40 ± 2.62
14	1.49	40	28.64	15	39.09	44.78 ± 0.80	339.73	369.49 ± 0.49
15	10	24.52	50	15	18.83	38.15 ± 0.25	313.5	299.94 ± 16.76
16	3.96	40	50	180	31.12	16.19 ± 0.13	256.67	238.86 ± 2.64
17	10	4	32.75	15	19.27	31.15 ± 0.57	313.23	304.45 ± 2.46
18	10	4	50	180	21.02	19.98 ± 0.62	249.10	239.59 ± 9.72
19	0.75	4	33.5	105.75	18.49	19.57 ± 0.27	243.04	247.00 ± 1.76
20	0.75	4	50	180	40.55	18.17 ± 1.13	230.67	243.97 ± 1.44
21	0.75	23.98	36.65	180	20.51	30.07 ± 0.56	257.33	259.49 ± 7.94
22	7.6	40	20	15	39.12	42.42 ± 0.26	337.68	359.75 ± 2.08
23	5.38	22	35	97.5	29.99	19.18 ± 0.84	257.25	226.34 ± 1.53
24	0.75	40	50	15	34.85	38.97 ± 1.00	348.78	347.46 ± 0.04
25	3.99	4	20	180	27.61	31.53 ± 0.23	302.27	295.15 ± 3.18
26	0.75	23.44	20	90.9	35.69	35.50 ± 0.60	314.30	318.41 ± 5.11
27	0.75	20.15	49.5	88.94	33.42	30.21 ± 1.56	269.75	268.40 ± 3.29

Response values are the mean of three replicates. TPC: total polyphenol content. GAE: gallic acid equivalent. TE: Trolox equivalents.

**Table 3:** Analysis of variance of the regression parameters of the total polyphenol yield and the antioxidant capacity of EAE of the pisco grape pomace.

Response	Y_1_: TPC					Y_2_: Antioxidant Capacity				
Source	Sum of Squares	df	Mean Square	F-Value	*p*-Value	Sum of Squares	df	Mean Square	F-Value	*p*-Value
Model	1537.99	8	192.25	53.48	<0.0001	38,917.57	8	4864.7	15	<0.0001
X_1_-Tannase	2.49	1	2.49	0.6939	0.4171	786.84	1	786.84	2.43	0.1367
X_2_-Cellulase	16.05	1	16.05	4.46	0.0507	479.19	1	479.19	1.48	0.2398
X_3_-Temperature	283.34	1	283.34	78.81	<0.0001	9094.98	1	9094.98	28.05	<0.0001
X_4_-Time	785.68	1	785.68	218.54	<0.0001	14,415.47	1	14,415.47	44.46	<0.0001
X_1_X_2_	-	-	-	-	-	3326.98	1	3326.98	10.26	0.0049
X_3_X_4_	107.12	1	107.12	29.8	<0.0001	1760.49	1	1760.49	5.43	0.0316
X_1_^2^	17.05	1	17.05	4.74	0.0447	-	-	-	-	-
X_3_^2^	228.65	1	228.65	63.6	<0.0001	2981.37	1	2981.37	9.19	0.0072
X_4_^2^	18.19	1	18.19	5.06	0.0389	2259.98	1	2259.98	6.97	0.0166
Residual	57.52	16	3.6			5836.62	18	324.26		
Lack of Fit	46.68	10	4.67	2.58	0.1287	3725.84	12	310.49	0.8826	0.5997
Pure Error	10.84	6	1.81			2110.78	6	351.8		
Cor Total	1595.51	24				44,754.19	26			
Fit Statistics										
R^2^	0.9639					0.8698				
R^2^ _Adjusted_	0.9456					0.8116				
R^2^ _Predicted_	0.9085					0.7067				
CV (%)	6.32					6.13				

TPC: total polyphenol content; *p*-values less than 0.05 indicate that model terms are significant.

**Table 4:** Box–Behnken experimental design for PLE of pisco grape pomace, polyphenolic yield of the extract, and antioxidant capacity.

	Independent Variables			Response Variables			
	X_1_	X_2_	X_3_	Y_3_		Y_4_	
Run	Ethanol Concentration (%)	Temperature (°C)	Extraction Cycles	TPC		Antioxidant Capacity by DPPH	
				(mg GAE g^−1^ dw)		(μmol TE g^−1^ dw)	
				Predicted	Experimental	Predicted	Experimental
1	90	120	3	41.51	40.05 ± 0.69	283.93	289.10 ± 2.43
2	90	80	1	33.69	34.07 ± 1.04	253.98	253.40 ± 2.38
3	50	80	3	43.27	41.85 ± 0.01	298.81	304.27 ± 6.51
4	50	40	1	32.61	31.53 ± 1.23	260.39	277.81 ± 2.67
5	50	80	3	43.27	44.18 ± 1.55	298.81	301.35 ± 12.50
6	50	80	3	43.27	39.90 ± 2.17	298.81	308.08 ± 15.48
7	90	40	3	33.49	34.19 ± 1.68	294.58	277.74 ± 2.98
8	90	80	5	33.16	33.54 ± 0.20	241.68	253.94 ± 2.26
9	10	80	5	13.08	12.70 ± 0.68	130.32	130.90 ± 8.03
10	50	40	5	34.47	33.38 ± 1.07	306.27	310.86 ± 4.57
11	10	40	3	4.38	5.84 ± 0.46	107.38	102.22 ± 4.55
12	10	80	1	9.1	8.72 ± 0.17	110.59	98.33 ± 4.73
13	50	120	5	49.13	50.21 ± 2.04	313.28	295.86 ± 7.22
14	50	80	3	43.27	45.49 ± 0.50	298.81	298.83 ± 13.37
15	50	120	1	47.55	48.64 ± 0.15	351.73	347.14 ± 8.95
16	50	80	3	43.27	44.94 ± 1.50	298.81	281.54 ± 9.17
17	10	120	3	25.95	25.25 ± 0.31	216.38	233.23 ± 6.13

Response values are the mean of three replicates. TPC: total polyphenol content. GAE: gallic acid equivalent. TE: Trolox equivalents.

**Table 5:** Analysis of variance of the regression parameters of the total polyphenol yield and the antioxidant capacity of PLE of pisco grape pomace.

Response	Y_1_: TPC					Y_2_: Antioxidant capacity				
Source	Sum of Squares	df	Mean Square	F-Value	*p*-Value	Sum of Squares	df	Mean Square	F-Value	*p*-Value
Model	2901.32	9	322.37	69.5	<0.0001	87,221.11	9	9691.23	34.06	<0.0001
X_1_-Ethanol concentration	997.7	1	997.7	215.09	<0.0001	32,448.78	1	32,448.78	114.05	<0.0001
X_2_-Temperature	438.23	1	438.23	94.47	<0.0001	4836.36	1	4836.36	17	0.0044
X_3_-Extraction cycles	5.9	1	5.9	1.27	0.2966	27.68	1	27.68	0.0973	0.7642
X_1_X_2_	45.9	1	45.9	9.9	0.0162	3579.03	1	3579.03	12.58	0.0094
X_1_X_3_	5.09	1	5.09	1.1	0.3299	256.48	1	256.48	0.9014	0.374
X_2_X_3_	0.0196	1	0.0196	0.0042	0.95	1777.89	1	1777.89	6.25	0.041
X_1_^2^	1335.71	1	1335.71	287.96	<0.0001	40,858.42	1	40,858.42	143.6	<0.0001
X_2_^2^	3.2	1	3.2	0.6894	0.4338	2688.04	1	2688.04	9.45	0.018
X_3_^2^	43.21	1	43.21	9.32	0.0185	1100	1	1100	3.87	0.09
Residual	32.47	7	4.64			1991.65	7	284.52		
Lack of Fit	10.55	3	3.52	0.6419	0.6272	1571.2	3	523.73	4.98	0.0774
Pure Error	21.92	4	5.48			420.45	4	105.11		
Cor Total	2933.79	16				89,212.76	16			
Fit Statistics										
R^2^	0.9889					0.9777				
R^2^ _Adjusted_	0.9747					0.9490				
R^2^ _Predicted_	0.9308					0.7180				
CV (%)	6.37					8.82				

TPC: total polyphenol content; *p*-values less than 0.05 indicate model terms are significant.

**Table 6:** Validation of enzymatic and pressurized liquid extractions.

			TPC (mg GAE g^−1^ dw)			Antioxidant Capacity (μmol ET g^−1^ dw)		
Extraction	Optimal Conditions		Value Predicted	Value Experimental	Error Percentage (%) *	Value Predicted	Value Experimental	Error Percentage (%) *
EAE	Tannase (U/mL)	0.75	41.51	38.49	7.34	370.52	342.47	7.57
	Cellulase (U/mL)	40						
	Time (min)	15						
	Temperature (°C)	20						
PLE	Ethanol concentration (%)	54						
	Temperature (°C)	113	50.66	50.03	1.24	331.84	371.00	11.80
	Extraction cycles	3						

* The error percentage (%) was calculated by comparing the experimental value to the predicted value. EAE: enzyme-assisted extraction; PLE: extraction by pressurized liquids; TPC: total polyphenol content.

**Table 7:** Characterization of optimal extracts.

	Extraction Methods	
Parameters	EAE	PLE
TPC (mg GAE g^−1^ dw) *	38.49 ± 0.99 ^b^	50.03 ± 0.58 ^a^
TFC (mg QE g^−1^ dw)	51.78 ± 1.62 ^b^	75.48 ± 2.12 ^a^
DPPH (μmol TE g^−1^ dw) *	342.47 ± 1.29 ^b^	371.00 ± 8.89 ^a^
ORAC (μmol TE g^−1^ dw)	1687.47 ± 4.66 ^b^	1931.39 ± 90.26 ^a^
Sugars (mg Glucose g^−1^ dw)	262.58 ± 1.83 ^b^	266.79 ± 0.02 ^a^
Phenolic compounds (mg g^−1^ dw)		
Gallic acid	0.14 ± 0.01 ^b^	0.23 ± 0.01 ^a^
Catechin	0.37 ± 0.02 ^b^	0.69 ± 0.00 ^a^
Epicatechin	0.40 ± 0.01 ^b^	0.61 ± 0.02 ^a^
4-Hydroxybenzoic	NQ	0.19 ± 0.00 ^a^
Quercetin-3-rutinoside hydrate	2.31 ± 0.06 ^b^	2.88 ± 0.03 ^a^
Quercetin-3-O-rhamnoside	0.09 ± 0.00 ^b^	0.12 ± 0.02 ^a^
Kaempferol	0.05 ± 0.00 ^b^	0.04 ± 0.00 ^a^

Values with different letters in the same row are significantly different (*p* < 0.05). TPC: total polyphenols content; TFC: total flavonoid content. EAE: enzyme-assisted extraction; PLE: extraction by pressurized liquids. NQ: not quantifiable. * Optimal validation values.

## Data Availability

The data used or analyzed during this current study are available from the corresponding author upon reasonable request.

## Supplementary Materials

The following supporting information can be downloaded at https://www.mdpi.com/article/10.3390/molecules30142977/s1, Figure S1: Correlation of TPC and antioxidant capacity of pisco grape pomace extracts.
