# Supplementary material for: Efficient Conditions of Enzyme-Assisted Extractions and Pressurized Liquids for Recovering Polyphenols with Antioxidant Capacity from Pisco Grape Pomace as a Sustainable Strategy
Source: Molecules. 2025 Jul 15;30(14):2977. doi: 10.3390/molecules30142977 (PMC12299454; doi:10.3390/molecules30142977)
Supplement: Supplementary file 1 [file molecules-30-02977-s001.zip › molecules-3727092-supplementary.pdf]

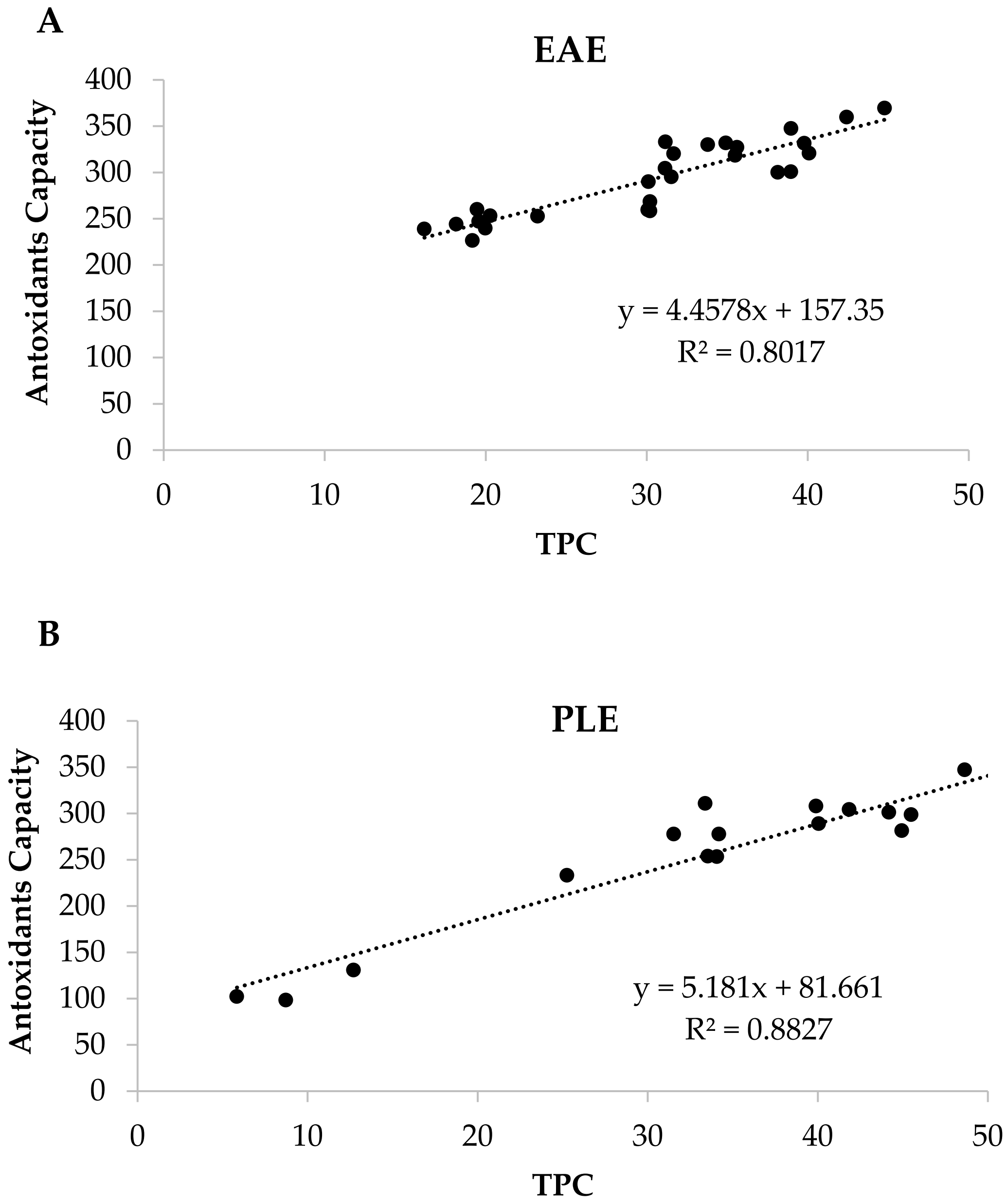

S1. Correlation of TPC and antioxidant capacity of pisco grape pomace extracts. TPC: Total polyphenol content. A) EAE: Enzyme-assisted extraction. B) PLE: Pressurized liquid extraction.
